# “Game on!” Teaching gamification principles for library instruction to health sciences information professionals using interactive, low-tech activities and design-thinking modalities

**DOI:** 10.5195/jmla.2019.636

**Published:** 2019-10-01

**Authors:** Nicole Capdarest-Arest, Eugenia Opuda, Rachel Keiko Stark

**Affiliations:** Head, Blaisdell Medical Library, University of California, Davis, CA, ncapdarest@ucdavis.edu; Dimond Library, University of New Hampshire, Durham, NH, eugenia.opuda@unh.edu; Library, Sacramento State University, Sacramento, CA, stark@csus.edu

## Abstract

**Background:**

Gamification is correlated with increased motivation and engagement of learners and is increasingly being incorporated into library instruction. Opportunities for librarians to learn and practice principles of gamification can be helpful for those desiring to incorporate gamification into instruction. This report describes the development and delivery of an interactive special content session at MLA ’18, the 2018 Medical Library Association annual meeting in Atlanta, Georgia, focusing on principles of low-tech game design for health sciences library classroom instruction.

**Case Presentation:**

The special content session, titled “Design, Play, Learn: A Special Content Session to Design a Game for Database Instruction,” was designed and delivered using multimodal instruction (e.g., flipped classroom, didactic component, active learning) that also incorporated principles of design thinking. A pre- and post-survey was given to all participants at the beginning and end of the session to measure confidence and desire to incorporate gamification into instruction and as a formative feedback indicator for instructors. Participants reported increased confidence and desire to use games for library instruction after participating in the session. A selection of games was uploaded to a shared content folder designed for course participants as an ongoing repository for ideas and communication.

**Conclusions:**

For librarians who are interested in incorporating principles of gamification into library instruction, attending a relatively short hands-on workshop can facilitate learning and confidence around prototyping and creating games for use in library instruction. We intend to improve upon the workshop and offer it again in additional contexts, based on direct observations of the session and participant feedback.

## BACKGROUND

Gamification in education is correlated with learners’ increased motivation and engagement in the learning process [1]. Many gamification studies in the higher education and health sciences literature highlight general principles of gamification and introduce examples of using serious, high-tech games (e.g., digital, computerized, or online games; video or virtual reality games) for classroom instruction. Moreover, there is a growing trend of libraries utilizing or designing high-tech games for library instruction, orientation, and training [2, 3]. Often using or designing high-tech games requires a number of additional resources including time, money, and digital programming skills, which presents challenges in the feasibility of game design for many instruction librarians.

While the idea of gamification in education conjures images of interactive mobile games and virtual reality, low-tech games (e.g., analog or non-digital games, board games) provide value to both instructors designing the game and students playing the game through a level of accessibility that is not always found in high-tech games and a level of face-to-face collaboration that builds interpersonal experience. When designing games, librarians often lack the necessary resources to create high-tech games, such as coding skills, time to learn how to use technology to create games, or funding to hire coders to build a game. Furthermore, students may lack the right devices that support high-tech gaming and may experience a steep learning curve to play high-tech games.

Prior studies on low-tech gamification in libraries [4, 5] focus on the tactile nature of low-tech gaming as a way to meaningfully engage students in library instruction and encourage collaborative learning. Previous research also indicates that using low-tech approaches for scavenger hunts allows libraries with limited staffing to engage students in gamification [4]. Low-tech games allow librarians to more quickly experiment with game design with fewer resources and lower stakes, while students only need the game pieces and rules in order to play.

Design thinking, an approach to creative problem solving, combines the processes of identifying problems, brainstorming solutions, and prototyping those solutions [6]. Designing educational games to increase knowledge around topics related to health sciences library instruction can be challenging and overwhelming. The phased process of design thinking—empathize, define, ideate, prototype, test—works well to innovate and test new ideas, such as game creation. The application of design thinking in game design for library instruction settings allows librarians to identify common problems in health sciences library instruction, brainstorm solutions through the form of games, develop prototypes of games, and test the games in order to adapt and refine the games to solve the initial problems.

This report describes the development and delivery of an interactive special content session at the 2018 Medical Library Association (MLA) annual meeting (MLA ’18) that focused on the principles of low-tech game design for library classroom instruction in the health sciences. Informed by the principles of low-tech gamification and design thinking, the authors created an interactive teaching session for health sciences librarians to experience designing, creating, and incorporating low-tech games into instructional activities. The session included several components designed to engage and prepare the health sciences librarian attendees: (1) suggested readings to be conducted prior to the MLA ’18 session; (2) an icebreaker activity for attendees to introduce themselves; (3) a short, didactic lecture delivered by the presenters; (4) a sample low-tech library instruction game, developed by the presenters, for attendees to build on or adapt; and (5) an interactive, team-based activity wherein participants used a design-thinking process to develop their own prototype game.

We distributed pre-session and post-session surveys to all participants at the beginning and end of the session to gauge participants’ comfort with and likelihood of incorporating gamification concepts that they learned in the session at their own institutions. In this report, we describe the development and delivery of the session, the results from the pre- and post-session survey, and lessons learned and ideas for future work related to gamification for library instruction.

## CASE PRESENTATION

The special content session, titled “Design, Play, Learn: A Special Content Session to Design a Game for Database Instruction,” was held at MLA ’18 in Atlanta, Georgia. The session was relatively informal, meeting attendees were able to drop in and out of the session, and no sign ups were required. The session was ninety minutes long, with most of the time being devoted to hands-on game design.

Prior to designing the elements of the interactive teaching session, we identified several outcomes for participants attending the session: (1) leave with a basic understanding of game design, (2) explore ideas for how to incorporate low-tech games into library instruction, (3) experience collaboratively designing and building a low-tech game, (4) explore ideas for assessing games and gamified instruction, and (5) develop components of a game, or an idea for a game, to utilize during library instruction. To prepare to meet these outcomes, we created a shared document of suggested readings on gamification, outlined a presentation covering background information about gamification, and prepared to facilitate the exploration of game-design during the special content session. Additionally, we created an ice-breaker activity to facilitate participant introductions, as well as pre- and post-session surveys.

We strived to highlight accessibility as a core value of “Design, Play, Learn” by focusing on low-tech gamification and using tactile materials that are easily purchased in any convenience or office supply store. All items created in preparation for the session—including the presentation, suggested readings, sample game, and assessment survey—are available in a public Google Drive folder <http://Bit.ly/MLAGameDesign>, which was shared with all of the special content session participants.

### Pre-session preparation

We collaboratively generated a list of suggested readings for participants to access during and after the special content session. The readings included several articles with general information about games and gamification in education, articles describing specific examples of applying low-tech gamification in health sciences library instruction, books focusing on game development, and articles on assessing gamification as an educational approach.

To supplement the list of suggested readings, we also created a short presentation for the session using a free online presentation tool, Mentimeter. The presentation covered the session outline, session outcomes and expectations, a behavior agreement, information about sharing and using the presentation materials, background information about using games in education, and instructions for the hands-on activities. Via the session expectation slide, we wanted to explicitly communicate that participants would not be expected to design and complete a game by the end of the ninety-minute session and that the goal was to experience game design in a safe environment and to leave the session with ideas about game design basics and library-related applications. The behavior agreement emphasized the value of respectful interaction and responsible behavior during the session to establish an open and inclusive session experience for all participants. Additionally, we encouraged the open sharing and use of “Design, Play, Learn” materials and provided this information to participants during the presentation.

### Design of the sample game

We created a low-tech card game prior to the special content session to provide attendees with an example game to build on or to use as a reference to design a different game. The intended audience for the sample game was health sciences students learning about library database searching. The game was designed to help students practice making search strings and adapting them to the databases available at the students’ institutions. Through game play, students could practice developing relevant database search strings using Boolean operator cards and health-specific terminology cards. After several rounds of editing, we tested the game with other librarians and students to identify any issues with the instructions and game play.

### Pre- and post-session assessment

After designing the presentation and creating the sample game, we designed an anonymous, five-item, pre- and post-session survey (supplemental appendix) with the primary goal of garnering formative feedback for future delivery of the session and for reviewing participants’ experience with gamification, participants’ confidence with gamification, and participants’ plans for future use of gamification in instruction sessions.

As the special content session was designed to be active and allow participants to learn through doing, the survey was intentionally short and utilized a five-point Likert scale. Items in the survey included: (1) “I have experience with gamification in a library instruction setting”; (2) “I feel confident using gamification in a library instruction setting”; (3) “I plan to use what I learned in this class in my future instruction sessions”; (4) “I plan to use the game from this session, or one I built during this session, in my future instruction sessions”; and (5) “I plan to assess gamification when I use it in my future instruction sessions.”

### “Design, Play, Learn”: the MLA special content session

The “Design, Play, Learn” session was held in a large ballroom in the meeting hotel with round tables to facilitate group work. Attendees were not required to sign up prior to the session, which allowed people to filter in later and leave early. We placed a variety of supplies including pens, markers, colorful paper, index cards, dice, rubber bands, scissors, and paper clips on all of the tables so that participants could utilize various low-tech and accessible tools to design their games. Pre-session surveys, copies of the sample game instructions, and cards for the ice-breaker activity were also included on the tables.

At the start of the session, all attendees were asked to fill out the five-item, pre-session survey. One of the presenters then collected the surveys and stored them away in a file folder. During the session, we wanted to ensure that participants could utilize most of the ninety minutes for hands-on game design. In the first fifteen minutes, we provided a short presentation on gamification that included definitions of gamification, the list of suggested gamification readings, and examples of our experiences with low-tech gamification in libraries. The presentation also included a link to the shared Google Drive folder that participants could access after the presentation to view all of the presentation materials. Attendees then conducted a short ice-breaker activity with cards to introduce themselves to their tables and share how much experience they each had with gamification in library instruction.

To utilize active-learning pedagogy for the remainder of the session, participants (in self-selected groups of two to eight) were instructed to physically create a game that followed the rules and design of the sample game or to design their own games using the provided tools and supplies. We wanted to ensure that the participants left the session with a game that would be interesting to their students and that could be utilized in a future library instruction session.

For the remainder of the session, participants practiced designing their own games or building on the sample game. They created game pieces with the supplies at their tables, wrote descriptions and rules for their games, and took photos of their work. The presenters walked around the room during the hands-on activity to answer questions, provide feedback on game designs, and serve as game-testers. We encouraged attendees to upload their game photos and instructions into the shared Google Drive folder so that others could view their game designs and adapt them under a Creative Commons license, similar to how we shared the game and presentation during the session.

At the end of the session, participants filled out the post-session survey, which they left on their tables to be collected. Additionally, participants were encouraged to provide verbal feedback to the presenters after the session. We hope to solicit more information from participants who provided their contact information at the end of the special content session for follow up. Unfortunately, we could not count the number of participants who left and came in throughout the session and are unable to provide an accurate return rate on the surveys.

## RESULTS AND FEEDBACK

**Figure 1:**
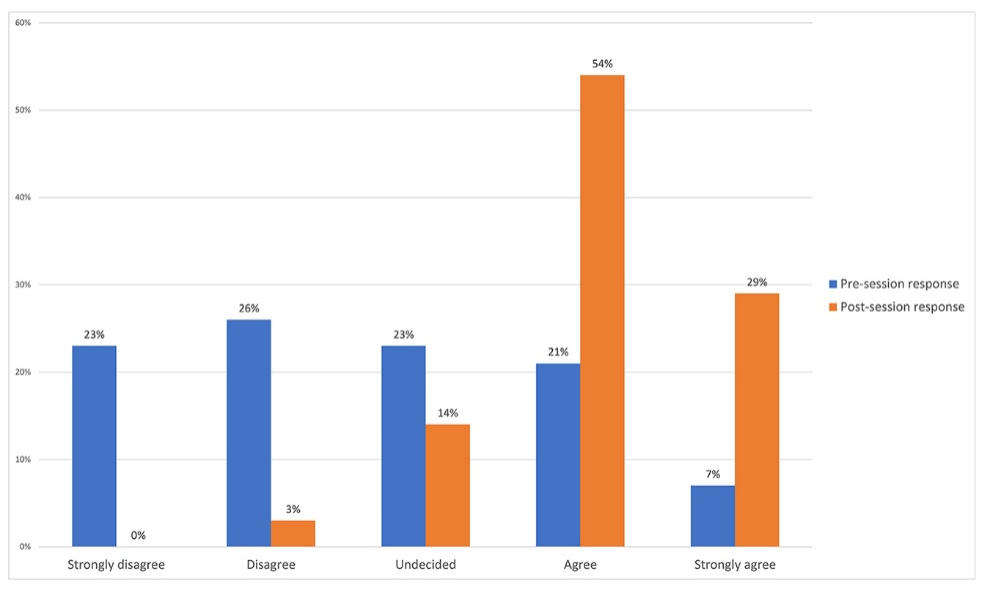
Pre-session versus post-session responses to survey question: “I feel confident using gamification in a library instruction setting.”

A total of 43 session participants completed the pre-session survey, and 35 completed the post-session survey. Changes in aggregate proportions of answers were noted between the pre- and post-session surveys. Before participating in the session, 12/43 (28%) of respondents reported feeling confident using gamification in a library instruction setting; after participating in the session, 29/35 (83%) of respondents reported feeling confident using gamification in a library instruction setting (Figure 1).

**Figure 2:**
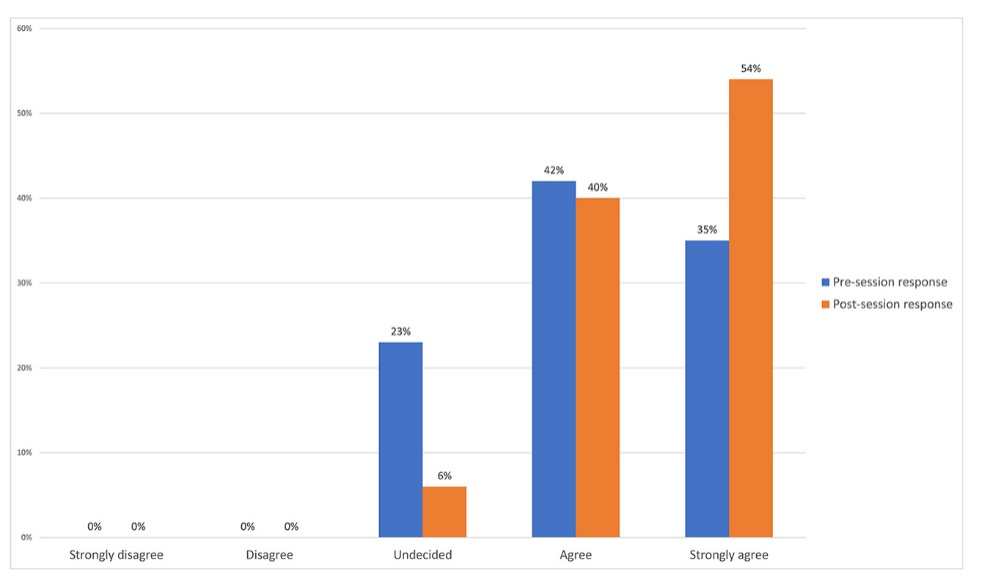
Pre-session versus post-session responses to survey question: “I plan to use what I learned in this class in my future instruction sessions.”

Responses related to the likelihood of whether participants planned to use what they learned during the session in their future instruction classes also increased, with 77% of pre-session survey respondents stating that they would incorporate learning from the session into their own instruction sessions and 94% of post-session survey respondents stating that they would (Figure 2). Similarly, when asked whether they planned to use the game from this session (i.e., the sample game developed by the instructors) or one they built on their own during the session in future instruction, 46% of pre-session survey respondents indicated they were likely or highly likely to do so, with that percentage increasing to 86% of post-session survey respondents.

In addition, we received unsolicited verbal and written feedback (via email or handwritten on the post-session surveys) regarding impressions of the session. Selected redacted comments (indicated by “XX”) to protect anonymity included:

“Design, Play, Learn: A Special Content Session to Design a Game for Database Instruction” was one of the best sessions I attended at MLA. My colleague and I are now planning our instruction for a course in the fall semester.

This was one of the most helpful sessions I attended at MLA.

As a result of the game seminar, I am using a space XX has...to deliver staff education on XX, XX and XX.

Playing through your created game would have helped or hearing examples of games created. I will check out the [shared folder] for ideas, but I wanted it in the session.

We have collaboration ideas to continue what we started [during the session]!

This could have easily been a 2-part session with the 1st part where we play a game and the 2nd part where we design one (like the one in this session). Maybe a future MLA [continuing education] CE?? Excellent session!!

This session really helped me make connections with colleagues for future collaborations. I really loved this session.

## DISCUSSION

Overall feedback from the “Design, Play, Learn” special content session was positive and indicated that the short session was helpful for many attendees to experience low-tech game design and to increase their confidence in incorporating more interactive teaching elements to their library instruction. Coupled with the qualitative feedback outlined above, findings from this session echoed that of others’ with regard to engagement and motivation [1]. As we did not require attendees to sign up for the session in advance, attendees could join and leave the session at their own will, and we chose not to attempt to enforce an attendance policy that would contradict the meeting set up.

The ebb and flow of audience member traffic for the duration of the session meant that the number of total pre- and post-session surveys collected were difficult to track. Should we run this session again, we would endeavor to set a scenario to better control for attendance and an additional follow-up assessment. Nonetheless, through formal and informal feedback gathering, we were impressed that some attendees were able to create ready-to-play games by the end of the session that attendees could take back to their home institutions to implement.

We identified a number of unexpected benefits from the special content session based on the interactive and hands-on approach to the session. Within a short amount of time, participants’ confidence levels with incorporating more interactive teaching elements into library instruction increased. Active prototyping and game testing in a safe and experimental environment encouraged participants to collaboratively exercise creativity and problem-solving skills. The interactive nature of the session also helped to foster connections between participants and facilitated networking that some attendees indicated would last after the session itself. Running the session in a game-like way using a design-thinking approach echoed others’ findings related to increasing engagement, motivation, and creativity with regard to gamified or design thinking–focused sessions [1, 5, 6]. Coupling the design-thinking approach with game-based learning is, we believe, a novel approach that our team hopes to explore more and expand upon in the future.

Future directions that we would take for this special content session include: (1) building in more time for participants to debrief and share their work, (2) allowing participants to play the sample game that the presenters designed prior to designing a game themselves, and (3) splitting the session into two parts: one focused on game design and the other focused on assessment. While a ninety-minute session was enough to build participants’ confidence, having extended session time to share attendee game designs and to explore gamification assessment will facilitate stronger participant engagement, strengthen participant confidence in utilizing games, and better demonstrate the impact of gamification in library instruction.

## SUPPLEMENTAL FILE

Appendix Pre/post evaluation

## 

**Nicole Capdarest-Arest, AHIP**, ncapdarest@ucdavis.edu, https://orcid.org/0000-0002-0174-5587, Head, Blaisdell Medical Library, University of California, Davis, CA

**Eugenia Opuda**, eugenia.opuda@unh.edu, Dimond Library, University of New Hampshire, Durham, NH

**Rachel Keiko Stark, AHIP**, stark@csus.edu, Library, Sacramento State University, Sacramento, CA
