# Supplementary material for: “Game on!” Teaching gamification principles for library instruction to health sciences information professionals using interactive, low-tech activities and design-thinking modalities
Source: J Med Libr Assoc. 2019 Oct 1;107(4):566–71. doi: 10.5195/jmla.2019.636 (PMC6774538; doi:10.5195/jmla.2019.636)
Supplement: Appendix [file jmla-107-566-s001.pdf]

Journal of the Medical Library Association

Supplemental content to
J Med Libr Assoc. Oct;107(4):dx.doi.org/10.5195/jmla.2019.636
www.jmla.mlanet.org
© Capdarest-Arest, Opuda, Stark 2019

"Game on!" Teaching gamification principles for library instruction to health sciences information professionals using interactive, low-tech activities and design-thinking modalities

Nicole Capdarest-Arest, AHIP; Eugenia Opuda; Rachel Keiko Stark, AHIP

APPENDIX

Pre/post evaluation

1. I have experience with gamification in a library instruction setting

Strongly disagree	Disagree	Undecided	Agree	Strongly agree
1	2	3	4	5

2. I feel confident using gamification in a library instruction setting

Strongly disagree	Disagree	Undecided	Agree	Strongly agree
1	2	3	4	5

3. I plan to use what I learned in this class in my future instruction sessions

Strongly disagree	Disagree	Undecided	Agree	Strongly agree
1	2	3	4	5

4. I plan to use the game from this session, or one I built during this session, in my future instruction sessions

Strongly disagree	Disagree	Undecided	Agree	Strongly agree
1	2	3	4	5

5. I plan to assess gamification when I use it in my future instruction sessions

Strongly disagree	Disagree	Undecided	Agree	Strongly agree
1	2	3	4	5
